# Measurement of Telomere Length in Colorectal Cancers for Improved Molecular Diagnosis

**DOI:** 10.3390/ijms18091871

**Published:** 2017-08-29

**Authors:** Eric Le Balc’h, Nathalie Grandin, Marie-Véronique Demattei, Serge Guyétant, Anne Tallet, Jean-Christophe Pagès, Mehdi Ouaissi, Thierry Lecomte, Michel Charbonneau

**Affiliations:** 1 CHRU Hôpital de Tours Trousseau, avenue de la République, 37170 Chambray-lès-Tours, France; ericlebalch85@gmail.com (E.L.B.); guyetant@univ-tours.fr (S.G.); anne.tallet@univ-tours.fr (A.T.); jean.pages@univ-tours.fr (J.-C.P.); mehdi.ouaissi@univ-tours.fr (M.O.); thierry.lecomte@univ-tours.fr (T.L.); 2 UMR CNRS 7292, UFR Pharmacy, University of Tours, Parc Grandmont, 31 avenue Monge, 37200 Tours, France; nathalie.grandin@uca.fr (N.G.); baud@univ-tours.fr (M.-V.D.)

**Keywords:** colorectal cancer, telomere length, *KRAS* mutations, telomere restriction fragment analysis

## Abstract

All tumors have in common to reactivate a telomere maintenance mechanism to allow for unlimited proliferation. On the other hand, genetic instability found in some tumors can result from the loss of telomeres. Here, we measured telomere length in colorectal cancers (CRCs) using TRF (Telomere Restriction Fragment) analysis. Telomeric DNA content was also quantified as the ratio of total telomeric (TTAGGG) sequences over that of the invariable Alu sequences. In most of the 125 CRCs analyzed, there was a significant diminution in telomere length compared with that in control healthy tissue. Only 34 tumors exhibited no telomere erosion and, in some cases, a slight telomere lengthening. Telomere length did not correlate with age, gender, tumor stage, tumor localization or stage of tumor differentiation. In addition, while telomere length did not correlate with the presence of a mutation in *BRAF* (V-raf murine sarcoma viral oncogene homolog B), *PIK3CA* (phosphatidylinositol 3-kinase catalytic subunit), or MSI status, it was significantly associated with the occurrence of a mutation in *KRAS*. Interestingly, we found that the shorter the telomeres in healthy tissue of a patient, the larger an increase in telomere length in the tumor. Our study points to the existence of two types of CRCs based on telomere length and reveals that telomere length in healthy tissue might influence telomere maintenance mechanisms in the tumor.

## 1. Introduction

Colorectal cancer (CRC) is the second leading cause of cancer-related death in Europe [1]. Most colorectal cancers arise through genetic instability of the genome. A genetic model of colorectal carcinogenesis that was proposed around 15 years ago remains a paradigm in studies on solid tumor progression [2]. However, despite progress in the understanding of the molecular background of these cancers, effective molecular classification remains a challenge. Microsatellite instability (MSI), chromosomal instability (CIN) as well as CpG island methylator phenotype (CIMP) seem to play a major role in these cancers [1,3]. Consequently, most of the classifications adopted to date are based on MSI, CIN and CIMP and attempt, in addition, to correlate these with the presence of mutations in *KRAS* (Kirsten rat sarcoma viral oncogene homolog) or *BRAF* (V-raf murine sarcoma viral oncogene homolog B) [1,4]. Mutational inactivation of *APC* (adenomatous polyposis coli), a tumor suppressor gene, is considered one of the earliest events in CRC tumorigenesis and is followed by the occurrence of activating mutations in *KRAS*. Microsatellites are short repetitive DNA sequences that are subject to frame-shift mutations and base-pair substitutions during DNA replication and are involved in DNA repair. MSI, or instability of microsatellite sequences, is related to defects in the mismatch repair system of DNA (known as MMR system) and these defects, initially associated with Lynch syndrome, generate two distinct types of phenotypes referred to as MSI-high (MSI-H) and MSI-low (MSI-L) [1]. Loss of *MLH1* (mutS homolog 1) function, for instance, originally associated with Lynch syndrome, also occurs in 15% to 20% of sporadic CRCs. Lynch syndrome is the result of germline mutations in one of the alleles of MMR genes (mainly *MSH2* (MutS protein homolog 2) and *MLH1* genes). The somatic mutation of the second allele leads to an alteration of the MMR system and the appearance of mutations leading to a carcinogenesis process. In sporadic CRCs, the cause is the hypermethylation of the two alleles of the *MLH1* gene. MSI-H accounts for around 22% of CRC cases [5]. Many studies have considered MSI status as a major prognostic biomarker because, for instance, MSI-H is associated with increased survival [1]. CIN refers to genome rearrangements consisting of abnormal chromosome complement or number. Tumors with CIN account for around 60% of CRC cases [6].

The linear chromosomes of eukaryotic organisms require particular protection at their extremities. Telomeres represent the ends of these linear chromosomes; they contain repeated TG-rich sequences not coding for proteins. Telomeric DNA sequences specifically associate with specialized proteins such as, for instance in vertebrates, proteins of the shelterin complex. These telomeric proteins provide protection of the telomeric ends, as they prevent their erosion and degradation [7]. Thus, telomeres protect chromosome ends from DNA repair activities that reseal chromosome internal DNA breaks occurring during DNA damage [8]. It is now currently admitted that telomere dysfunction is at the origin of a number of degenerative disorders and that these telomere syndromes predispose to cancer [9]. In vertebrates, shelterin is a complex of six telomeric proteins (TRF1 (Telomere Repeat-binding Factor 1), TRF2 (Telomere Repeat-binding Factor 2), POT1 (Protection of Telomeres protein 1), TIN2 (TRF1-Interacting nuclear Factor 2), TPP1/PIP1 (Telomere Protection Protein 1/POT1-Interacting Protein 1) and RAP1 (Repressor/Activator site-binding Protein 1) that prevent inappropriate recombination and fusion between telomeres, and also have major roles in telomere replication and length regulation. In recent years, the human shelterin complex and in particular one its components, TRF2, have been found to play a major role in cancer biology [9,10]. Thus, *TRF2*, but also *TRF1* and *TIN2*, are overexpressed in certain cancers and, in addition, several mutations in genes encoding the components of the shelterin complex have been identified in cancers [11,12]. Moreover, at the telomeres, the DNA damage response and cell cycle control machineries, which are altered in cancer cells, are regulated by the shelterin complex proteins [8]. Finally, TRF2 and RAP1 also bind to internal telomere sequences and modulate transcription of a number of genes, some of which might be potentially important to the survival of cancer cells [11,12].

Telomere sequences naturally erode with ongoing cell divisions, due to intrinsic mechanisms associated with the fixed 5′ to 3′ polarity of replication of the DNA of the genome. Below a certain threshold, shortened telomeres result in a DNA damage-induced cell cycle arrest, which is the equivalent of replicative senescence in cultured cells. By limiting the replicative potential of cells, telomere length serves as “biological clock” and telomere erosion acts as a barrier against tumorigenesis in healthy tissue. Paradoxically, telomere erosion or telomere dysfunction also induces chromosomal instability and favors the emergence of tumors [13]. However, following cancer initiation, tumor cells must overcome the telomere-controlled replicative senescence barrier, and all, without exception, have an absolute need for maintaining functional telomeres to sustain continuous and unlimited cell proliferation. In around 90% of cancer types, this occurs through up-regulation of telomerase, a reverse transcriptase with a built-in RNA template specialized in telomeric DNA replication that is naturally repressed in most somatic tissues [14]. In the remaining 10% of cancer types, an alternative pathway called the ALT (Alternative Lengthening of Telomeres) pathway, functioning either by amplifying telomere sequences by homologous recombination or by sister chromatid exchange is used [15,16].

Measurements of telomere length have been reported in several studies on human CRCs during the past 25 years or so (see, for instance, [17,18,19]). In the present study, we measured telomere length in 125 CRCs using TRF (Telomere Restriction Fragment) Southern-blot analysis [20]. There are currently several methods starting with genomic DNA for measuring telomere length in cells, among which TRF analysis, qPCR (quantitative Polymerase Chain Reaction) amplification of telomere repeats relative to a single copy gene), PCR-based single telomere length analysis (STELA) and flow FISH (fluorescent in situ hybridization of telomere repeats in individual cells or chromosomes) are the most common [21]. Among these methods, TRF analysis is often considered as the gold standard [21]. One of the advantages of TRF analysis is to obtain an absolute value for telomere length in kilobases (Kb). Tumor cells that have positive telomerase activity or normal somatic cells have a telomere length of 5–10 Kb, while tumor cells that have developed the ALT pathway have long (>20 Kb) and heterogeneous (0.5–50 Kb) telomeres as compared to the shorter and more homogeneous telomeres of telomerase positive tumor cells [16]. In addition, 3D techniques based on 3D telomere fluorescence in situ hybridization followed by quantitative analysis have allowed analyzing telomere organization in the nucleus throughout the cell cycle [22,23]. It was reported that specific 3D nuclear telomeric profiles were associated with cancer and allowed for the identification of patient subgroups [22,24].

In the present study, we have measured telomere length in 125 CRC tumors and in adjacent healthy tissue using TRF analysis. Then, we have investigated the possible links between telomere maintenance in CRC and some clinical, anatomopathological and molecular parameters used in current practice. This study is an explorative one, with no pre-specified hypotheses and, therefore, there was no independent validation cohort.

## 2. Results

### 2.1. Description of the Tumors Analyzed

A total of 135 patients suffering from CRC were included in the present study, among which 125 provided enough tumoral DNA for telomere length/telomeric DNA content analyses. The cohort analyzed for telomere maintenance parameters comprised 69 (55.2%) men and 56 (44.8%) women. Mean patient age was 72 years (standard deviation 11.9). Right and transverse colon were the sites for the primary tumor in 50 (40.0%) of the cases, while left colon and rectum were the primary sites in 70 (56.0%) and 5 (4.0%) cases, respectively. The distribution of tumor localization according to tumor stage is presented in Table 1. The distribution of mutations in the *KRAS*, *BRAF* and *PIK3CA* genes, as well as the occurrence of microsatellite instability (MSI), depending on tumor stage is given in Table 2.

### 2.2. Telomeres Are Globally Much Shorter in Tumors Than in Healthy Tissue

**Figure 1:**
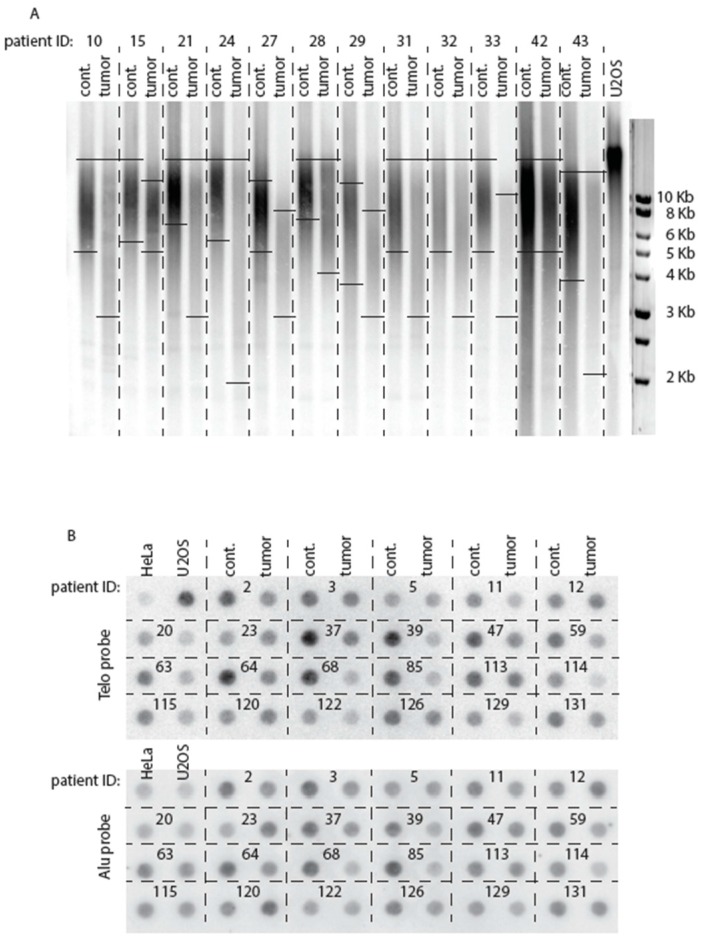
(**A**) Representative Southern blots of measurements of telomere length in DNA extracted from tumors (right lanes of each patient for which patient ID (identification) number is the same as that shown in Tables S1 and S2), together with DNA extracted from adjacent healthy tissue (left lanes) using TRF analysis (3–5 μg per sample) and a ^32^P-labeled (TTAGGG)_3_ telomeric probe. The center of the smear of labeled telomeric sequences was used to assess a telomeric mean value in kilobases (Kb) of DNA. Note that the length of the smear is also important because it reflects the degree of scattering of all telomere lengths around the mean value of the whole cell population. Horizontal full lines within the figure indicate the upper and lower limits of these smears. Telomere length in U2OS, an osteosarcoma-derived cell line, was measured under identical conditions. In both (A) and (B) panels “cont.” means “control”; (**B**) quantification of total telomeric DNA content in patient tumors (and their control adjacent healthy tissue) by dot blot analysis using the so-called Telo/Alu method. The indicated tumor and control DNAs (10 ng per sample) were hybridized to a ^32^P-labeled (TTAGGG)_3_ telomeric probe (top panel), then to a ^32^P-labeled Alu probe (bottom panel) that was used to normalize the telomeric signal to the total amount of genomic DNA. Telo/Alu ratio was also measured for HeLa cells (which are telomerase positive), as well as for U2OS, a prototype of ALT positive cells. ALT cells have much longer telomeres than telomerase positive cells, hence the stronger Telo signal in the former, serving as a positive control. All 125 samples were processed similarly, but only some of them are illustrated here.

Telomere length was measured by TRF analysis (Figure 1A) and, in addition, the amount of telomeric sequences, TTAGGG, was quantified by measuring the TTAGGG sequences/Alu sequences ratio (Figure 1B). Mean telomere length was significantly smaller in the CRC samples compared to adjacent healthy mucosa samples (7.2 Kb in tumors samples vs. 9.7 Kb in adjacent mucosa; *n* = 125; *p* < 0.0001; Table S1). When considered stage by stage (TNM classification), telomere length in the tumors was also significantly smaller than in the healthy surrounding tissue (Table 3). On the other hand, the total amount of telomeric sequences, determined as the TTAGGG sequences/Alu sequences ratio is given for each tumor in the column “percent of telomeric DNA content tumor vs. cont.” of Table S2. We arbitrarily defined 80% as being the threshold above which tumors were considered as exhibiting either no telomere erosion or at most moderate telomere erosion because of TRF-measured telomere length. Not taking 100% as the actual expected number indicating absence of telomere erosion resulted from the fact that we observed that 100% did not always correlate with actual maintenance of telomere length in the tumor. Hence, this decision to lower this threshold to 80% to evaluate more accurately the correlation between Telo/Alu measurement and TRF measurement (Table S2). Raw data of representative examples are also presented in Figure 1B. Diminution of the Telo/Alu sequences ratio in most of the colorectal tumors analyzed here confirmed the concomitant telomere shortening measured by TRF analysis (see Section 2.4).

Mean telomere length did not vary between the different tumoral TNM classification stages (6.8 Kb for stage I, 7.6 Kb for stage II, 7.2 Kb for stage III and 6.8 Kb for stage IV; *p* = 0.7). In addition, there was an absence of correlation between telomere length and the presence or absence of metastasis (7.3 Kb vs. 6.8 Kb; *p* = 0.3), or tumor location (*p* = 0.6).

### 2.3. A Class of Colorectal Cancers Does Not Exhibit Telomere Erosion

In practice, there were several ways to try to discern different classes of tumors based on the size of their telomeres. One way of doing it was to apply an arbitrarily defined value for telomere length and then class tumors as being either above or below this value. We could have taken, for instance, the mean value of telomere length of the 125 healthy tissues as that threshold numerical value. However, this was not satisfying, as telomere length in healthy tissue can greatly vary from one patient to another. Furthermore, we reasoned that it would be biologically more sounded to compare telomere length in a given tumor with telomere length in healthy tissue from the same patient. This could potentially help to explain the importance of telomere length deregulation in the tumor. We therefore defined the event of telomere erosion as our leading criterion to establish two classes of CRC tumors: those exhibiting clear telomere erosion and those with either no apparent change in telomere length or even slight telomere elongation. Separating “no change in telomere length tumors” and “longer telomeres tumors” in two distinct groups would have led to too few cases for each group, thus diminishing the impact of the statistical analysis. These two classes were therefore arbitrarily grouped together. In addition, doing this way was technically more reliable, since it is very difficult to appreciate the mean value of telomere length on the Southern blots due to their very heterogeneous lengths (Figure 1A). On the other hand, defining a tumor as exhibiting telomere erosion or not is much easier, because, in most cases, this event is readily discernable by eye.

Out of the 125 tumors analyzed by TRF, 91 exhibited telomeres that were shorter than in the healthy surrounding tissue taken from the same patient, while in the remaining 34 patients telomere length in the tumor was either roughly identical to that in the normal tissue or slightly larger (Figure 1A; Table S1). In fact, five out of 34 only of these tumors belonging to the later class actually exhibited elongated telomeres (tumors #3, #40, #58, #88 and #90; Table S2). In the rest of this study, we arbitrarily used these two classes of tumors to conduct additional statistical tests. In the 91 tumor samples with shortened telomeres, mean telomere length was statistically significantly shorter than in their normal adjacent tissue (6.5 Kb in the tumors versus 10.0 Kb in the normal surrounding tissue; *p* < 0.0001; Table 4). In the 34 tumors with elongated telomeres or telomeres of the same length as the control, mean telomere length was 9.2 Kb versus 8.8 Kb in the normal adjacent tissue, not significantly different (*p* = 0.50; Table 4).

### 2.4. Comparison of Telomere Restriction Fragment and Telo/Alu Methods to Measure Telomere Length

**Figure 2:**
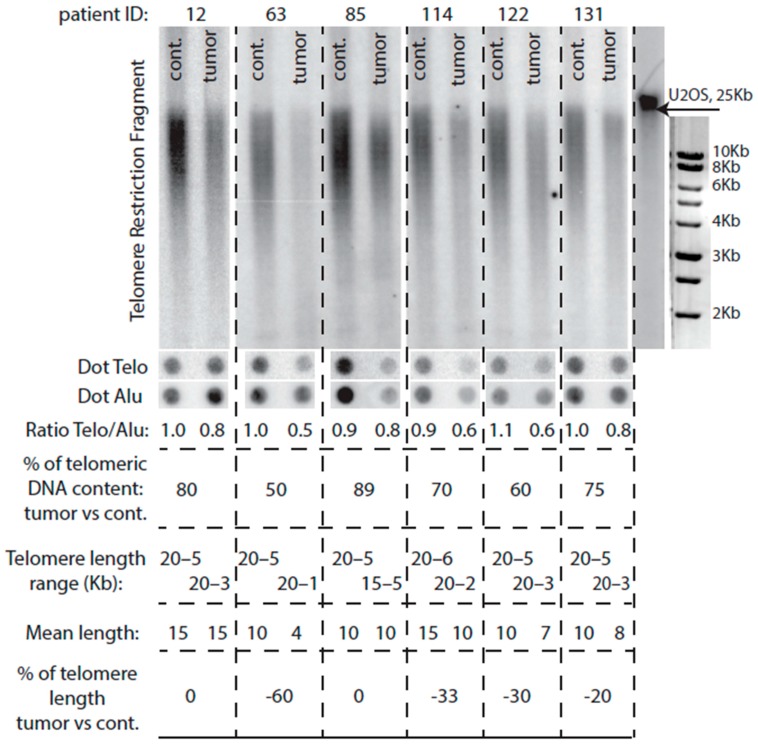
Comparisons between the two methods used in the present study to evaluate telomere length. Top panels illustrate telomere length measurement by TRF analysis (as explained in the legend to Figure 1), while middle panels illustrate, in the same tumors, the quantification of the total amount of telomeric sequences by the Telo/Alu method (as described above in the legend to Figure 1); “patient ID” is as defined in the legend to Figure 1). All four bottom panels show the indicated parameters associated with each tumor DNA and its control (“cont.”).

We note that among the 34 tumors with either slightly elongated telomeres or telomeres roughly of the same size as in healthy tissue, as measured by TRF analysis, only 18 simultaneously exhibited an increased Telo/Alu ratio, meaning that 16 tumors had telomeres equal or larger than in control but a Telo/Alu ratio <80%, the threshold chosen for absence of telomere erosion in the tumor, as defined above (Figure 2; Table S2). Conversely, 24 other tumors that exhibited Telo/Alu ratio >80% (compared with control tissue) did not have, by TRF analysis, telomeres longer than that of the healthy control tissue, meaning that 67/91 tumors that had eroded telomeres also exhibited Telo/Alu ratio <80% (Table S2). These results suggest that, although measurement of the total amount of telomeric sequences by the Telo/Alu ratio method does not always reflect the presence of long telomeres, it nevertheless remains that the vast majority of tumors with eroded telomeres, 67/91, also exhibited a dramatically decreased amount ot total telomeric sequences.

### 2.5. Mutated KRAS Preferentially Associates with Short Telomeres

The 91 tumor samples with significantly eroded telomeres were next compared to the 34 tumor samples with good telomere maintenance for additional clinical parameters. There was no significant difference in mean telomere length between the two groups concerning age (*p* = 0.3), gender (*p* = 0.8), tumor location (*p* = 0.3), stage (CRC TNM classification) (*p* = 0.8), tumor differentiation stage (*p* = 0.9) or degree of lymphocytic infiltration (*p* = 0.5). Interestingly, we observed significantly less cases with mutated *KRAS* status in the 34 samples with good telomere maintenance than in the 91 samples with eroded telomeres (~17% vs. ~39%, respectively; *p* < 0.05), the amount of mutated *KRAS* in the total population (*n* = 125) being ~34%. On the other hand, there was no difference between the two groups concerning the occurrence of mutations in *BRAF* (*p* = 0.7), *PIK3CA* (*p* = 0.7) or the MSI status (*p* = 0.6).

### 2.6. Telomere Lengthening in the Tumor Preferentially Occurs in Patients with Short Telomeres

**Figure 3:**
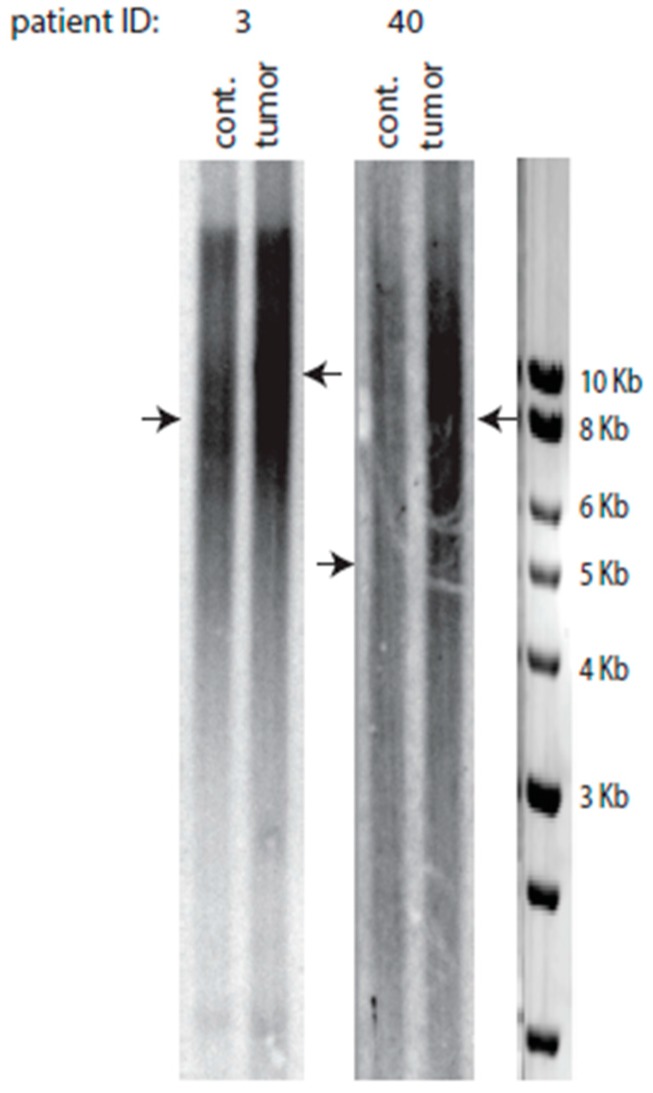
Comparisons between telomere length pattern in a tumor (right lane for each patient) and in its surrounding healthy tissue (“cont.” stands for control, left lane for each patient; “patient ID” is as defined in the legend to Figure 1.) concerning two patients exhibiting tumors with elongated telomeres. Arrows indicate the position of the mean value of telomere length for each sample.

Interestingly, too, telomeres in the healthy control tissues of the 34 tumors exhibiting telomeres equal in size or larger than in control were significantly shorter than their control counterparts in the 91 patients with eroded telomeres (8.8 Kb for the former vs. 10.0 Kb for the latter, *p* = 0.021); thus, the shorter the telomeres in healthy tissue of a patient, the larger an increase in telomere length in the tumor. This observation may suggest that the mechanisms of cancer-initiated telomere lengthening might differ depending on the actual size of the telomeres of the healthy cells prior to cancer initiation (see Discussion). When we looked into more detail at the profiles of the TRF Southern blots, we observed that, in some cases, telomeres in the control (healthy tissue) samples of the patients with tumors with telomeres equal or larger than in control were more heterogeneous in size than telomeres in the corresponding tumor (Figure 3). The significance of this observation is not yet known.

### 2.7. Correlations between Telomere Length and Overall Survival

**Figure 4:**
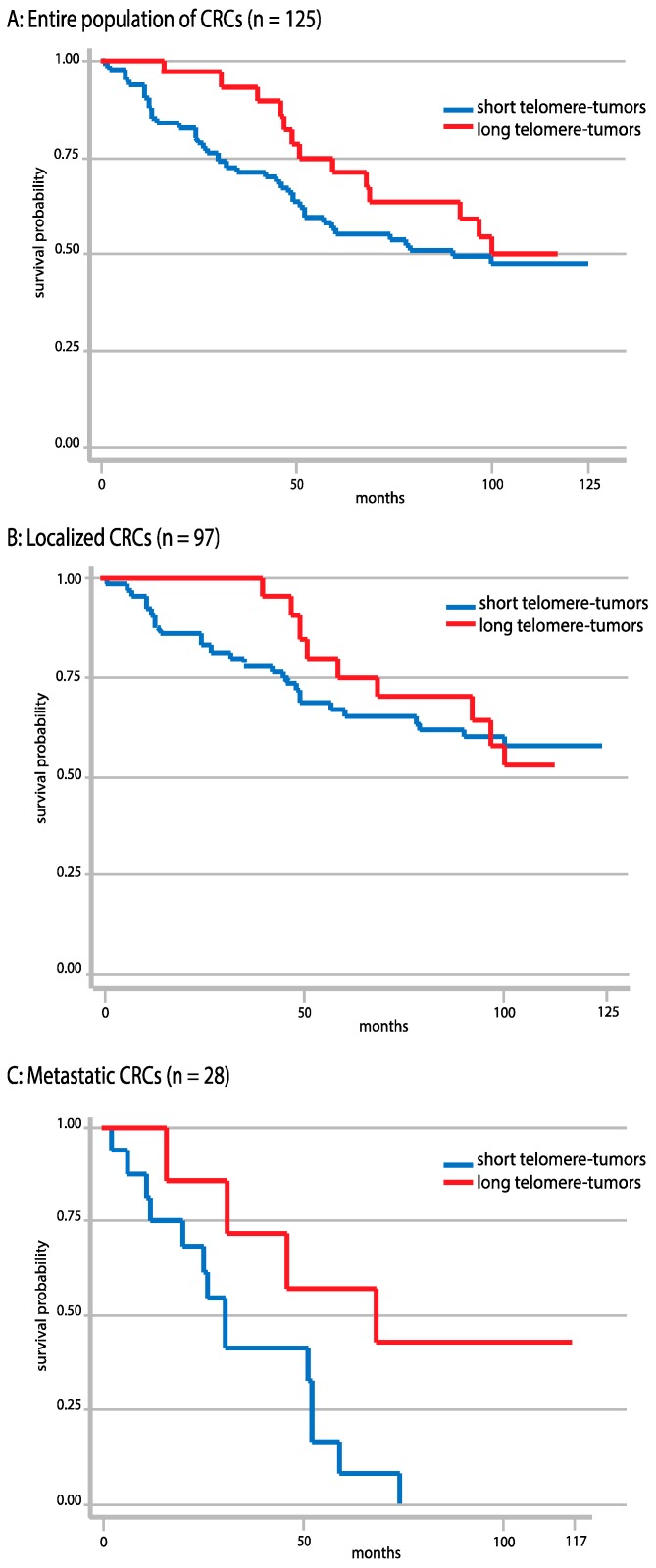
Evolution with time (in months) of overall survival (expressed as the probability of survival) comparing the two classes of CRCs arbitrarily defined based on their telomere length (with the long telomere-tumor cohort in red, and the short telomere-tumor cohort in blue): in the entire population of analyzed CRCs (**A**); that of localized CRCs (**B**) and that of metastatic CRCs (**C**). Note that the term “long telomere-tumors” was used here for simplicity, but that, in fact, it refers to “same size as controls or slightly elongated telomere-tumors”.

Possible correlations between overall survival and telomere length were assessed by comparing the evolution of survival (expressed as the probability of survival) in the two classes of tumors arbitrarily distinguished based on telomere length (Figure 4). The curve of survival in the 91 tumors with eroded telomeres was slightly more incurved than that of the 34 tumors exhibiting good telomere maintenance, meaning that patients with tumors with longer telomeres better survived than those with tumors with eroded telomeres (Figure 4). Thus, after 103 months of recording, ~52% of the patients with maintained length telomere-tumors were still alive, while a similar percentage of survival, ~53%, was achieved after only 60 months in the patients with short telomere-tumors; the percentage of survival attained in this class of patients after 103 months being ~43% only. Overall survival was significantly correlated with telomere length in metastatic tumors (*n* = 28) in univariate analysis (*p* = 0.03), but not in multivariate analysis. However, there was no statistically significant difference in overall survival between the maintained length telomere-tumor class and the short telomere-tumor class when the entire population of analyzed CRCs (*n* = 125) was taken into account (*p* = 0.36). It was also not the case when the population of localized CRCs (*n* = 97) was taken into account (*p* = 0.76).

### 2.8. Correlations between Telomere Length and Tumor Localization

We also observed that the group of 91 tumors with eroded telomeres contained 40.7% of cancers located in the right and transverse colon, while the group of 34 tumors with telomeres with maintained length, comprised 32.3% of right-colon and transverse colon cancers. In other words, 37/48 (77.1%) right-colon tumors had eroded telomeres, while 49/70 (70.0%) left-colon tumors had eroded telomeres. Therefore, right-colon cancers had more frequently eroded telomeres than telomeres with maintained length. On the other hand, right-colon and transverse cancers had telomeres with a mean value of 7.1 ± 2.1 Kb (*n* = 47), not significantly different (*p* = 0.65) from that in left-colon, sigmoid and rectal cancers (7.3 ± 2.3%, *n* = 78). Therefore, telomeres were not shorter in right-colon and transverse cancers than in CRCs located at other sites. In addition, right-colon and transverse tumors, on the one hand, as well as left-colon, and sigmoid tumors, on the other hand, had both significantly shorter telomeres than in the healthy surrounding tissue (Table 5). Note that rectal cancers were present at a very low rate in the analyzed cohort. In fact, almost all of the operated patients with rectal cancers did not fulfill one of the required criteria for inclusion into the cohort and were therefore excluded from the analysis (see Materials and Methods). Finally, we observed that telomeres were not shorter in MSI tumors (6.9 ± 1.8 Kb, *n* = 19) than in MSS tumors (7.3 ± 2.3 Kb, *n* = 105, the *p* value of the *t*-test being >0.05).

The relevant molecular characteristics of the 135 CRCs analyzed in the present study, as well as their telomeric parameters, are shown in Table S2.

## 3. Discussion

Previously, a number of studies have shown that telomere length was smaller in colorectal tumors than in adjacent healthy tissues [17,18,19,25,26,27,28,29,30,31,32,33,34,35]. The present data agree with these studies, establishing that, among the 125 CRCs in which telomere length was measured by TRF analysis, telomeres were significantly much shorter than in the healthy adjacent mucosa, 7.2 Kb in tumors samples versus 9.7 Kb in adjacent mucosa.

Interestingly, we were able to distinguish two classes of CRCs, one (91/125) exhibiting clear telomere erosion (6.5 Kb in the tumors vs. 10.0 Kb in the normal surrounding tissue), a second one (34/125 CRCs) with telomeres of the same length or slightly longer than those in the healthy tissue (9.2 Kb in the tumors vs. 8.8 Kb in healthy adjacent tissue). Previous studies have shown that only a few percent of tumors elongate their telomeres (5% to 14% depending on the studies) and the vast majority shortened or maintained them [18,19,29,33,36,37,38]. The finding that 5/125 of the CRC tumors analyzed here (4% of the cohort) exhibited telomere elongation is therefore not very different from the lower value found in previous studies. Our percentage of tumors with telomeres exhibiting maintained length may be somewhat smaller than from these previous findings because we regrouped in the same class the tumors that maintain telomere length constant and those that elongate their telomeres compared with the healthy adjacent tissue. The class we defined with eroded telomeres accounted for ~73% of the 125 CRCs analyzed, leaving ~27% of the tumors with unchanged or elongated telomeres.

The reasons for the existence of CRCs with either greatly eroded telomeres or, rather, telomeres maintained at a constant length or even slightly elongated are not known. Recent studies have started to provide an explanation to the presence of persistent telomere erosion in some types of cancer such as CRCs. *TRF2* overexpression has been observed in many tumors and might be at the origin of very high genome instability in these cells. By examining the length of individual telomeres in cells overexpressing *TRF2*, Nera et al. [39] were able to identify a subpopulation of termini that had undergone loss of almost the entire telomeric tract, as a consequence of *TRF2* overexpression-induced replication stalling. Moreover, 3D analysis of telomere organization combined with TRF2 immunofluorescence showed that some Hodgkin and Reed-Sternberg cells exhibited massive attrition of telomere sequences together with massive increase of TRF2 not associated with telomeres, while others experienced a massive loss of TRF2 signals physically linked to telomeres [40]. These observations underlie a major role for *TRF2* expression in telomere organization in tumor cells and, as a consequence, in the regulation of telomere length and genome stability in these cells.

Although TRF2 plays a major role in genome stability in tumor cells, to our knowledge, *TRF2* overexpression has not previously been reported to take place in CRCs. The present results provide an alternative explanation to the phenomenon of dramatic telomere erosion taking place in the great majority of CRCs. Indeed, we have observed here that in patients with tumors exhibiting constant length/slightly elongated telomeres, telomere length in the adjacent normal tissue was significantly smaller than that in the adjacent normal tissue of patients with tumors exhibiting eroded telomeres; therefore, the shorter the telomeres in healthy tissue of a patient, the larger an increase in telomere length in the tumor. Several possibilities arise to explain this observation, among which the most likely is that when healthy cells become tumoral, the efficiency of the mechanisms of telomere maintenance that have been activated concomitant with cell transformation is proportional to telomere length. Indeed, in the newly transformed cell, the intensive cell proliferation needed for tumor progression is going to rapidly erode telomeres. If the newly transformed cell has relatively short telomeres, then cell division-associated telomere erosion is rapidly going to become prejudicial to the cell, leading, as a solution to this problem, to an improvement in the efficiency of the telomere maintenance mechanisms (likely via up-regulation of telomerase in this type of cancer). As a result, telomeres are going to maintain telomere length initially present in the non-tumoral cell, even possibly elongating telomeres. If, on the other hand, the newly transformed cell has relatively long telomeres, telomere maintenance mechanisms are not going to be activated right away, efficient cell division still taking place due to the presence of long telomeres. It is only after telomeres have become critically short that telomerase will be activated. This scenario may well lead to a situation in which telomeres are always critically short, becoming re-elongated from time to time by irregular activation of telomerase.

In the present study, we also found that telomeres were not shorter in right-colon cancers than in tumors located in other sites, contrary to what has been reported previously [29,32]. On the other hand, right-colon cancers were found more frequently in the 91 tumors with eroded telomeres than in the group of 34 tumors with constant length/slightly elongated telomeres, although it should be noted that the numbers do not strikingly differ between the two groups, 40.7% vs. 32.3%, respectively. In addition, we observed that telomeres were not shorter in MSI tumors than in MSS tumors, contrary to what has been reported before [32]. We did not detect a significant difference in telomere length depending on tumor stage, contrary to a previous study reporting that telomere length in cancer tissue was significantly correlated with tumor stage, telomeres being longer in advanced tumors [18,19]. We do not have any explanation for this discrepancy, particularly as these two previous studies also used TRF analysis to assess telomere length [18,19].

Very few data are available concerning telomere characteristics and mutational status in CRC. When comparing the 91 tumors with eroded telomeres with the 34 tumors with maintained telomere length, we could not find statistically significant differences between the two groups concerning the mutational status of *BRAF*, *PIK3CA* and the MSS/MSI genes. However, interestingly, our study highlighted a statistically significant relationship between telomere length and *KRAS* mutation status. Indeed, ~39% of the tumors with eroded telomeres harbored a mutation in *KRAS*, while ~17% of the tumors with maintained telomere length (*p* < 0.05) harbored a *KRAS* mutation. Notably, it has been observed that activation of the Ras/Raf/MEK/Erk pathway upregulated telomerase activity by increasing *hTERT* transcription [41]. *EGFR* overexpression also led to telomerase activation by *h*TERT phosphorylation via the PI3K/AKT pathway [42]. *KRAS* mutation is a negative predictive biomarker for anti-EGFR therapy (cetuximab and panitumumab) in metastatic CRC [43]. In metastatic CRC with non-mutated *KRAS* status, a relationship between an elevated telomere length and a good response (inhibition of proliferation) to anti-EGFR has recently been observed, without, however, any relationship between *KRAS* status and telomere length [44]. It is currently totally unknown why CRCs with eroded telomeres more frequently harbor a mutation in *KRAS* than CRCs with maintained telomere length.

Our survival graphs suggested that patients with maintained length telomere-tumors survived better than patients with short telomere-tumors, although the difference was not statistically significant when the entire population of analyzed CRCs was taken into account. However, in the 28 metastatic tumors present in the study, telomere erosion was significantly associated with diminished survival. Such an observation is compatible with the hypothesis sometimes put forward that more aggressive tumors exhibit telomeres that are constantly critically short because their rapidly undergoing cell divisions require frequent availability of telomerase activity that cannot be always guaranteed by the cellular machinery. Recently, a distinct class of extremely short telomeres, called t-stumps, was discovered in transformed human cells containing active telomerase [45]. The aggressive metastatic CRC tumors found here to be associated with poor survival and which exhibited dramatic telomere erosion might correspond to the acquisition of these t-stumps. Recently, in glioblastomas, as well as in Hodgkin lymphomas, correlations were established between median survival and nuclear telomere architecture and it was proposed that nuclear telomere architecture might become a prognostic biomarker [46,47]. It would be interesting to know whether the association we observed here between survival and telomere erosion in metastatic CRCs could also be signaled by changes in the 3D nuclear organization of the telomeres.

Finally, we found that measurement, in CRCs, of the total amount of telomeric sequences by the Telo/Alu ratio method was not in all situations in agreement with telomere length measured by TRF analysis. However, globally, we found a good relationship between telomere length and total amount of telomeric sequences, as 67 tumors among the group of the 91 with eroded telomeres also exhibited a total amount of telomeric sequences below the threshold that is considered low (Telo/Alu ratio <80%) compared with total amount in adjacent healthy tissue. On the other hand, only 18 of the 34 tumors with constant length/slightly elongated telomeres, as measured by TRF analysis, also had a Telo/Alu ratio >80%. Therefore, although this assay is easier to perform, it is not advisable to use it in routine as a clinical test, at least for this type of cancer. The reasons for the observed discrepancy between the two methods could be due to the numerous genetic instabilities associated with CRC. Indeed, genomic instabilities can lead to the generation of so-called GCRs (Gross Chromosomal Rearrangements), which are prone, among other unwanted events, to the intervention of telomerase in an attempt to repair the broken chromosome [48]. If this were the case in the CRCs analyzed here, then the total amount of telomeric sequences could become increased, due to the inappropriate intervention of telomerase at endogenous genomic sites, without concomitant lengthening of the telomeres, hence the observed discrepancy, in some cases, between the two methods.

## 4. Materials and Methods

### 4.1. Patients and Tissue Collection

This study was approved by the ethics committees of the “Centre Hospitalier Régional Universitaire” (CHRU) of Tours (France). Tumor samples came from a cohort of CRCs collected in the pathology department of the University Hospital of Tours. The collection contains frozen and formalin-fixed, paraffin-embedded tumor material, as well as paired normal tissue; written informed consent was obtained from all patients. According to French laws and recommendations, the collection has been declared to the French Ministry of Scientific Research and is registered under N° DC-2008-308. We constituted an exploratory cohort of 135 selected patients who had undergone surgery for a CRC between January 2007 and May 2008 at the Trousseau hospital of Chambray-lès-Tours. Inclusion criteria were as follows. Patients had to be over 18 years old and have been surgically treated for a CRC. Obtaining individual written consent prior to use of each sample, as recommended by the institutional guidelines, was required. Patients were excluded from the analyzed cohort when there was either absence of healthy tissue adjacent to the resected specimen, absence of data on microsatellite instability or on one of the *KRAS*, *BRAF* or *PIK3CA* status, or when a neo-adjuvant treatment had been previously delivered. For each case studied, the percentage of tumor cells was evaluated by a pathologist on a frozen section of the tumor sample. Only samples containing at least 50% of tumor cells were selected for this study. The presence of normal stromal cells in the tumor sample was not taken into account. Finally, rectal cancers were almost all excluded from the study because most of them did not fulfill the required inclusion criterion of having not been administered a neo-adjuvant treatment.

Two samples of colorectal tissues, tumoral and adjacent non-tumoral, were recovered from patients following surgery, immediately snap-frozen individually and stored confidentially at −80 °C by the tumor bank of Tours CHRU. Normal tissue (mucosal and submucosal tissue) was selected by a pathologist on surgical colorectal resections, at least 5 cm distant from the tumor, and immediately frozen for biobanking. Like for the tumor samples, a microscopic examination of a frozen section was realized for each normal tissue sample. Patient characteristics (age, sex, segmental colonic tumor location, *KRAS* status, *BRAF* status, PI3K status, MSI status and histological data) were collected from the records for further analysis. In addition, an anatomopathological study of the tumor samples was conducted in order to provide information concerning tumor differentiation state, vascular invasion and degree of peritumoral lymphocytic infiltration. The classification used in the present clinical study was the TNM (Tumor-Node-Metastasis) classification reactualized in 2010 in the 7th edition of the “Cancer staging manual” edited by the American Joint Committee on Cancer [49].

### 4.2. Genomic DNA Extraction, Mutational Analysis and MLH1 Promoter Methylation

Genomic DNA was extracted from frozen tissue samples using the QIAamp DNA Mini Kit (Qiagen, Courtaboeuf, France) according to the manufacturer’s instructions. DNA was quantified in a spectrophotometer. The presence of mutations in *KRAS* exon 2, *BRAF* exon 15 and in the catalytic subunit of PI3K, *PIK3CA*, were evaluated by pyrosequencing (Pyromark platform^®^; Qiagen) following amplification by Polymerase Chain Reaction (PCR). MSI status was determined following PCR amplification from tumoral DNA analyses of six microsatellite markers, NR21, NR24, NR27, BAT25, BAT26 and CAT25. The tumor was considered MSI-High when three or more of these markers showed instability, while MSI-Low was defined as taking place when one or two of the six markers showed instability [50]. Sequencing analyses were performed using the Applied Biosystems^®^ 3130 Genetic Analyzer (Thermo Fisher Scientific, Illkirch, France,). In sporadic (non-familial) CRCs, MMR defect results from the silencing of *MLH1* transcription by cytosine methylation. Histological sections of tumors were first processed for immunodetection of MLH1. In tumors with a lack of MLH1 protein, *MLH1* promoter methylation status was then measured by real-time PCR following sodium bisulfite conversion of tumor DNA, as previously described [51].

### 4.3. Telomeric DNA Quantification by Dot Blot for Telomere Length Measurement

This so-called Telo/Alu quantification of total telomeric DNA was performed as described previously [52]. Briefly, 10 ng only of genomic DNA were hybridized, following transfer to a nylon membrane, with a ^32^P-labeled telomeric repeat (TTAGGG)_3_ probe, followed by hybridization with an Alu-specific (5′-GTGATCCGCCCGCCTCGGCCTCCCAAAGT-3′) ^32^P-labeled probe. Results were analyzed using a GE Storm phosphorimager (GE Healthcare Life Sciences, Vélizy, France) and the ImageQuant LAS 4000 software (GE Healthcare Life Sciences, Vélizy, France).

### 4.4. Telomere Length Measurement by Telomere Restriction Fragment (TRF) Southern Blot Analysis

Measurement of telomere length by TRF analysis [20] was performed as described previously [52]. Briefly, 3–5 μg of tumor genomic DNA were digested with *Rsa*I and *Hinf*I and separated in a 0.9% agarose gel prior to transfer and hybridization with a (TTAGGG)_3_^32^P-labeled telomeric probe. Telomere tracts appear as a broad band or “smear”, which represents the average length of most chromosomes. The smear is very heterogeneous because telomere length not only varies between chromosome ends, but also between cells. Results were analyzed using a GE Storm phosphorimager and the ImageGauge software version 4.0 (Fujifilm, Denver, CO, USA).

### 4.5. Statistical Analysis

Statistical analyses were performed using standard tests, using the Stata 9.2 software (Stata Corp. LLC, College Station, TX, USA). To compare independent quantitative data, we used the Student’s *t*-test. To compare independent nominal qualitative data, Chi-square distribution was determined. To report overall survival curves, we used the Kaplan–Meier method, and log-rank test was used to compare them. A Cox proportional-hazard model was used to assess overall survival differences between patients with different telomere lengths. Modeling included the examination of associations between overall survival and clinically relevant variables. A *p* < 0.05 value was used as the significant threshold. All the data collected were anonymized and stocked in an Excel file.

## Figures and Tables

**Table 1:** Distribution of tumor localization according to tumor stage in the 125 tumors in which telomere length was measured.

Stage	Right and Transverse Colon	Left and Sigmoid Colon	Rectum	Total
Stage I	2 (1.6%)	6 (4.8%)	3 (2.4%)	11 (8.8%)
Stage II	15 (12.0%)	28 (22.4%)	1 (0.8%)	44 (35.2%)
Stage III	18 (14.4%)	23 (18.4%)	1 (0.8%)	42 (33.6%)
Stage IV	15 (12.0%)	13 (10.4%)	0 (0%)	28 (22.4%)
Total	50 (40.0%)	70 (56.0%)	5 (4.0%)	125 (100%)

**Table 2:** Distribution of mutations in *KRAS* (Kirsten rat sarcoma viral oncogene homolog), *BRAF* (V-raf murine sarcoma viral oncogene homolog B) and *PIK3CA* (phosphatidylinositol 3-kinase catalytic subunit), and occurrence of microsatellite instability (MSI), as a function of tumor stage in the 125 tumors in which telomere length was measured.

Gene Mutations	Stage I	Stage II	Stage III	Stage IV	Total
*KRAS* mutated	2	12	15	13	42 (33.6%)
*BRAF* mutated	0	8	8	1	17 (13.6%)
*PIK3CA* mutated	0	4	3	6	13 (10.4%)
MSI ^a^	0	9	7	3	19 (15.2%)

^a^: Among the 19 tumors with MSI, one only (of stage IV) exhibited MSI-Low status, the other 18 tumors being MSI-High.

**Table 3:** Comparison of telomere length in tumor and healthy adjacent tissue in the different CRC (colorectal cancer) stages.

Parameters	Stage I	Stage II	Stage III	Stage IV
Number of cases	11	44	42	28
Telomere length in tumor tissue	6.8 Kb	7.6 Kb	7.2 Kb	6.8 Kb
Telomere length in adjacent healthy tissue	10.0 Kb	9.7 Kb	10.3 Kb	8.6 Kb
Significance	*p* = 0.01	*p* < 0.0001	*p* < 0.0001	*p* < 0.001

**Table 4:** *p* Values of Student *t*-test ^a^ comparing telomere lengths (in kilobases) in the 34 tumors-group and the 91 tumors-group and in their respective adjacent healthy tissues.

Tumor and Control Classes	91 Control (Average, *n* = 91) 10.0 ± 2.8	91 Tumor Group ^b^ (Average, *n* = 91) 6.5 ± 1.7	34 Control (Average, *n* = 34) 8.8 ± 2.4	34 Tumor Group ^c^ (Average, *n* = 34) 9.2 ± 2.2	125 Control (Average, *n* = 125) 9.7 ± 2.7	Tumor (Average, *n* = 125) 7.2 ± 2.2
Control (*n* = 91)		<0.0001	0.021		0.391	
Tumor (*n* = 91)	<0.0001			<0.0001		0.008
Control (*n* = 34)	0.021			0.50	0.075	
Tumor (*n* = 34)		<0.0001	0.50			3.947
Control (*n* = 125)						<0.0001
Tumor (*n* = 125)					<0.0001	

^a^: *p* values of Student *t*-test were performed in Microsoft Excel and were considered as statistically significant when <0.05; ^b^: The 91 tumor group contained the 91 tumors in which telomere length was found, by TRF analysis, to be smaller than in healthy adjacent tissue; ^c^: The 34 tumor group contained the 34 tumors in which telomeres were measured, by TRF analysis, to be either of the same length or larger than in the healthy adjacent tissue.

**Table 5:** Telomere length according to tumor localization and associated *p* values of *t*-test ^a^.

Parameters	Right and Transverse Colon	Left and Sigmoid Colon	Rectum
Number of cases	50	70	5
Telomere length in tumor tissue	7.0 Kb	7.3 Kb	7.1 Kb
Telomere length in adjacent healthy tissue	9.9 Kb	9.5 Kb	10.5 Kb
Significance	*p* < 0.0001	*p* < 0.0001	*p* = 0.07 ^b^

^a^: *p* values of Student *t*-test were performed in Microsoft Excel and were considered as statistically significant when <0.05; ^b^: Not statistically significant.

## Supplementary Materials

Supplementary materials can be found at www.mdpi.com/1422-0067/18/9/1871/s1.
