# Supplementary material for: Measurement of Telomere Length in Colorectal Cancers for Improved Molecular Diagnosis
Source: Int J Mol Sci. 2017 Aug 29;18(9):1871. doi: 10.3390/ijms18091871 (PMC5618520; doi:10.3390/ijms18091871)
Supplement: Supplementary file 1 [file ijms-18-01871-s001.zip › ijms-218673-supplementary/ijms-218673 suppl.TS1.pdf]

Table S1. Telomere mean lengths [a] and ranges in the 135 tumors and their respective healthy adjacent tissue (cont.) [b]

n°	Telomere mean length cont.	Telomere mean length tumor	Range of telomere length cont.	Range of telomere length tumor
1	N/A [c]	N/A	N/A	N/A
2	10	8	20-5	20-3
3	8	10	20-5	20-5
4	7	6	20-5	20-3
5	10	8	20-5	20-3
6	9	8	20-6	20-5
7	8	7	20-6	20-3
8	10	6	20-7	15-3
9	8	5	20-6	15-2
10	8	5	20-5	20-3
11	15	8	20-5	20-3
12	15	15	20-5	20-3
13	8	N/A	15-5	N/A
14	15	7	20-7	15-3
15	8	7	20-6	15-5
16	10	N/A	20-6	N/A
17	7	7	20-5	20-5
18	10	10	20-5	20-5
19	15	4	20-9	15-3
20	15	10	20-6	20-2
21	10	8	20-7	20-3
22	9	7	20-5	20-3
23	10	8	20-6	20-3
24	15	5	20-6	20-2
25	N/A	N/A	N/A	N/A
26	10	6	20-5	20-3
27	8	5	10-5	8-3
28	15	15	20-8	20-4
29	7	4	10-4	8-3
30	10	6	20-5	20-3
31	7	4	20-5	20-3
32	6	5	20-5	20-3
33	8	4	20-5	10-3
34	15	8	20-6	20-3
35	10	10	20-6	20-7
36	18	15	20-10	20-7
37	10	10	20-8	20-8
38	10	5	20-5	8-3
39	15	5	20-8	20-3
40	5	8	20-5	20-4
41	10	6	20-5	20-4
42	10	8	20-5	20-5
43	7	5	15-4	15-2
44	8	N/A	10-6	N/A
45	10	10	20-8	20-8
46	10	10	20-8	20-8
47	10	7	20-6	20-3
48	N/A	N/A	N/A	N/A
49	15	5	20-6	20-3

50	8	8	20-5	15-7
51	8	5	20-5	15-3
52	8	6	20-4	20-4
53	N/A	N/A	N/A	N/A
54	8	4	20-4	10-2
55	10	6	20-5	20-3
56	10	4	20-8	20-3
57	6	6	20-4	20-4
58	7	8	20-4	20-6
59	10	6	20-8	20-3
60	10	6	20-5	10-3
61	10	8	20-6	20-4
62	7	6	20-5	20-3
63	10	4	20-5	20-1
64	15	10	20-8	20-4
65	9	5	20-5	20-3
66	7	5	20-5	20-3
67	10	10	20-7	20-7
68	10	8	20-6	20-3
69	10	6	20-6	15-3
70	10	4	20-6	20-3
71	10	9	20-6	20-4
72	7	7	20-5	10-6
73	15	10	20-8	20-3
74	8	N/A	15-4	N/A
75	7	6	20-5	20-3
76	8	8	20-5	20-5
77	8	6	20-5	10-4
78	8	6	20-5	20-3
79	7	7	20-5	20-5
80	7	7	20-4	20-4
81	7	5	20-5	20-3
82	8	6	20-5	20-4
83	8	8	20-8	20-5
84	10	8	20-8	20-6
85	10	10	20-5	15-7
86	8	6	20-8	20-5
87	7	6	10-4	10-4
88	5	10	10-4	20-8
89	8	7	20-6	20-5
90	8	10	10-5	10-5
91	8	6	20-5	20-4
92	8	5	15-6	10-3
93	8	6	15-5	10-4
94	8	N/A	20-5	N/A
95	8	6	20-5	10-4
96	8	6	20-6	10-4
97	8	8	20-4	20-4
98	8	7	20-6	10-4
99	15	7	20-5	20-3
100	8	8	20-5	20-5
101	7	6	10-5	10-3
102	8	6	10-4	10-4
103	9	9	20-7	20-7
104	10	10	20-7	20-7
105	15	5	20-7	20-3
106	9	9	20-7	20-7

107	9	6	20-7	20-4
108	10	8	20-6	20-4
109	8	N/A	20-4	N/A
110	7	6	10-5	10-4
111	15	15	20-5	20-5
112	15	6	20-6	15-6
113	10	10	20-5	20-4
114	15	10	20-5	20-2
115	10	5	20-6	20-2
116	10	9	20-6	20-5
117	6	6	20-4	20-4
118	7	6	20-5	20-3
119	8	6	20-6	10-4
120	10	7	20-5	20-3
121	15	7	20-8	20-4
122	10	7	20-5	20-3
123	15	10	20-8	20-6
124	7	7	20-5	20-4
125	8	6	20-6	20-4
126	10	10	20-6	20-6
127	15	7	20-6	10-3
128	10	8	20-6	20-4
129	8	8	20-6	20-3
130	8	5	20-5	20-3
131	10	8	20-5	20-3
132	10	8	20-5	20-4
133	8	8	20-5	20-5
134	10	7	20-6	20-3
135	10	6	20-5	20-5

[a]: Telomere length, expressed in kilobases (Kb) was measured by TRF (Telomere Restriction Fragment) analysis. Mean values; represented size in Kb in the middle of the smear (see Fig. 1) while ranges indicate the upper and bottom limits of the smear; [b]: Tumors in which mean telomere length was larger than that in the corresponding control (5) are written over blue background, while those in which tumor telomere length was approximately equal to that in the healthy surrounding tissue (29) are written in red over yellow background; [c]: N/A stands for Non Applicable.
